# Reliability of the Balance Error Scoring System test is maintained during remote administration

**DOI:** 10.2217/cnc-2022-0006

**Published:** 2023-02-16

**Authors:** Joey Planchet, Camden R Lynch, Pamella L Mozzer, Daniel Seichepine

**Affiliations:** 1 Life Sciences Department, Neuropsychology Laboratory, University of New Hampshire, 88 Commercial Street, Manchester, NH 03101, USA

**Keywords:** balance, baseline, BESS, COVID, mTBI, remote, remote administration, remote assessment, sports, telehealth

## Abstract

**Aim:**

This study investigates if scores on the Balance Error Scoring System (BESS) are affected when administered remotely.

**Materials & methods:**

Participants included 26 undergraduate students, aged 19-32 (mean: 21.85 ± 2.95). Each participant received the BESS test remotely and in person, and scores on each were compared. To minimize potential practice effects, participants were randomly assigned to two equal sized groups to take the BESS remotely first or in person first.

**Results:**

The mean difference between scores for the remote and in-person assessments was 0.711 (95% CI: 0.708–2.131). There was no significant difference between scores (p = 0.312) indicating the BESS maintains reliability when administered remotely.

**Conclusion:**

Administration of the BESS remotely was possible without any significant challenges.

## Plain language summary

This article explains the results of an experiment using a balance test known as the Balance Error Scoring System (BESS). The BESS is used to diagnose concussions by giving it to athletes prior to an injury. If they get injured, the test is given again to find any worsening of balance which can suggest a concussion. Normally this test is done in person. This experiment tests if the scores are affected when you give the BESS test over Zoom. To figure this out, we gave the BESS test to people over Zoom and in person to see if their scores were different. We found that their scores were the same, meaning the BESS test can be given remotely without affecting its scores.

Mild traumatic brain injuries ([mTBIs]; commonly known as concussions) occur when direct or indirect forces to the head or body cause alterations in neuronal functioning [1]. These injuries can result in observable signs, as well as cognitive, emotional and physical symptoms such as balance problems and dizziness [2]. A clinical diagnosis of mTBI is made when there is loss of consciousness for less than 30 min, post-traumatic amnesia lasting less than 24 h and normal neuroimaging [3]. It is estimated that the majority of mTBIs go unreported and undiagnosed [4]; thereby making population-wide data unavailable. Participation in sports is a common cause of mTBIs. According to the Youth Risk Behavior Survey distributed by the CDC, it is estimated that about 15% of public and private high school students experience at least one sports or physical activity related concussion yearly [5]. Due to the prevalence of observable mTBIs in sports, prevalence data are regularly collected from this population. Outside of sports, clinical tests used to assess mTBIs are usually conducted after an injury which complicates the interpretation of results because the level of functioning before the injury is unknown. In a sports setting, baseline evaluations are often performed to facilitate the interpretation of these tests. An example of an easily observable and measurable symptom of mTBI is balance impairment [1], which can be detected by comparing baseline performance and postinjury performance to detect changes caused by head injury.

A commonly used diagnostic tool in the sports field or related practices is the Balance Error Scoring System (BESS) [6,7]; this is an objective and quantitative measure of postural stability used to assess mTBIs [8]. Among concussed and fatigued populations, this tool was shown to have moderate to good reliability, moderate to high criterion validity and high content validity [9]. The BESS is used within sports settings as a diagnostic tool to detect balance impairments secondary to an mTBI. Preseason baseline scores can be compared with scores obtained after an injury to determine the extent of balance impairment. Within this setting, the BESS is sensitive to mTBI related impairments up to 5 days after an injury [10]. The BESS or modified BESS (mBESS) is included in the concussion protocols of multiple major athletic associations including the National Collegiate Athletic Association (NCAA), the National Football League (NFL) and the Fédération Internationale de Football Association (FIFA) [11–13]. Additionally, the 2017 international Concussion in Sport Group recommended the implementation of the Sports Concussion Assessment Tool, 5th edition (SCAT-5), which uses the mBESS, to be used in athletic organizations for baseline, acute injury and serial evaluation [14]. The BESS is also used within research settings to investigate both short and long-term balance deficits related to mTBIs [15,16].

The COVID-19 pandemic has resulted in an increased reliance on remote services. Clinicians and researchers began offering remote alternatives to otherwise in-person visits to reduce pandemic related risks [17]. This shift revealed additional benefits of remote services, such as eliminating barriers caused by geographic distance and facilitating communication between providers and patients [18]. Conducting research remotely can also have many benefits, since improved access facilitates greater sample sizes.

Before administering traditionally face-to-face clinical or research services remotely, it is necessary to study the reliability of remote versions of these assessments. There are many potential issues that could interfere with their administration. During remote testing the physical environment of the participant cannot be as controlled, increasing the likelihood of interference. Internet and device complications can also interfere with the integrity of remote assessments. These technical issues must be considered when conducting services remotely. Types of technical issues can include poor camera and video resolution, poor audio quality, unreliable internet speed and connection, call drops and power outages. Extending an assessment to a remote modality is considered a significant modification and the added variables must be investigated alongside the original assessment before widespread application within both clinical and research settings.

While there has been a previous investigation of the feasibility of the mBESS when extended to remote use with a robot [19], there has yet to be a study to investigate the BESS itself and the issues with typical remote administration. The robot study used a shortened version of the BESS, had only six participants, did not address or describe technical errors and used technology not widely available. Additionally, the assessments were conducted postinjury using both a robot and an in-person clinician simultaneously. Our study used widely available technology and software (Zoom), that could be used in a wide range of research and clinical settings, analyzed technical errors that arose while using a counter-balanced design between in-person and remote administrations to investigate reliability. We also used a larger sample size of healthy participants. The aim of this study was to determine the consistency of remote and in-person scores on the BESS.

## Materials & methods

### Participants

A total of 26 undergraduate students between 18 and 40 years old participated during the fall of 2021 and spring of 2022. The university’s institutional review board granted approval for the study before beginning recruitment and data collection. Demographic information recorded included age, sex and years of education completed. Participants were informed of the full extent of their participation requirements for the study prior to obtaining their written consent. Participants were recruited through flyers placed on campus, announcements in classes, messaging and word of mouth. Each participant enrolled was administered the BESS twice, once face-to-face in the neuropsychology lab, and once remotely through Zoom. Additionally, participants were required to be fluent in English, have access to the internet, have someone who can act as a spotter during the remote assessment and have a device and hardware capable of streaming over Zoom.

### Study design

The BESS consists of three stances performed on both firm and foam surfaces. The stances include the double leg stance (feet together), single leg stance and tandem leg stance (heel to toe). Both the single leg and tandem leg stances require the nondominant foot in a specific position [20]. The dominant foot is identified by determining which foot is used to kick a ball. For the single leg stance, individuals stand on their nondominant foot, whereas for the tandem leg stance, the nondominant foot is placed heel to toe behind the dominant foot. For each of the three stances, balance is required to be maintained in each position for 20 seconds with eyes closed and hands placed on the hips. Errors include moving out of the testing position, opening the eyes, lifting the toes or the heels and taking one’s hands from the waist. Errors are recorded for each stance and aggregated into an overall score.

Two raters scored each assessment, and the average of the two scores were recorded as well as the individual rater’s score. Substantial practice in administrating and scoring the assessment was performed by each rater to ensure scoring synchronization prior to conducting the study. Half of the participants received the BESS remotely first and the other half were given the BESS in person first. The order in which participants took the remote and in-person assessments was determined randomly using Excel. Because aging effects have been observed with the assessment [8], participants were required to be between the ages of 18 and 40. Practice effects have also been observed in young adult populations [21] and; therefore, time between assessments could be no shorter than 24 h nor exceed 14 days.

For the remote assessment, participants were loaned a foam pad and a tape measure to measure out enough space for the assessment. The foam pad model used was the Airex Balance Pad™ with 50 × 41 × 6-cm-thick dimensions and medium-density foam. The testing area was required to be flat and 4 × 4 feet as ensured by the tape measure. Each remote assessment was administered by two researchers and the device used was connected to the lab’s internet to ensure the most reliable and consistent internet connection. Before the Zoom call, participants were emailed a link to https://www.speedtest.net to test their internet speeds before and after the assessment. They were then instructed to email these results to the researchers by taking a screenshot of the test results. Data recorded from the internet speed test included ping in milliseconds, download speed in megabits per second and upload speed in megabits per second. Participants were asked to indicate their operating system, type of device and model number, type of camera and model number as well as whether they were connected to the internet via Wi-Fi, ethernet or data. During the assessment, participants’ entire bodies were required to be in the frame so that the test could be administered properly.

Technical issues that arose during the video call were recorded by the researchers to determine whether they interfered with the overall balance scores. Types of technical issues included call drops, ‘freezing’, audio or video issues, outages and interruptions. The durations of the technical issues were assigned numeric values and were added upon completion of the assessment to establish an overall technical error score. Table 1 shows both the error types as well as their corresponding error values by duration. The severity of camera quality was characterized as being either slight, moderate, or extreme. Audio errors were put into subcategories based on if the audio became choppy, robotic or cut out. Call drops were not assessed on duration, and instead each instance was given a score of 5. Technical errors with higher severity and longer duration received higher error scores. Technical errors that were seen by researchers as not severe were not assigned a value. When a technical error happened during the balance test, the stance and surface in which it occurred was recorded as well. Because the remote portion of the assessment happened in a quasi-controlled environment, unforeseen interruptions were recorded as qualitative observations. After administering the remote assessment, researchers documented whether the technical issues significantly impacted administration or scoring. Researchers also documented which sections of the assessment were most affected by technical errors, if applicable.

**Table 1.** Table to calculate technical scores based off error type and duration.

Error type	Duration					Any instance
	1 s	1–5 s	5-10 s	10–15 s	15+ s	
Video freeze	1	2	3	4	5	–
Camera quality: slight pixelation	–	–	1	2	3	–
Camera quality: moderate pixelation	–	1	2	3	4	–
Camera quality: extreme pixelation	1	2	3	4	5	–
Audio issues: robotic	–	–	–	–	1	–
Audio issues: choppy	–	–	–	1	2	–
Audio issues: outage	–	1	2	3	4	–
Interruptions	1	2	3	4	5	–
Call drop	–	–	–	–	–	5 per instance

### Statistical analysis

A sample size estimation with power at 80% and alpha at 0.05 indicated that a sample size of 26 would be able to detect a 3-point difference in overall scores between assessments. Quantitative statistical analyses were performed for all variables. Both remote and in-person scores were compared to establish within-group differences by using a paired samples t-test with alpha set at .05. Additionally, the relation between overall in-person and remote scores were determined with a Spearman correlation coefficient. Paired samples t-test and independent samples t-test were used on the other variables when appropriate. Additionally, technical complications that come from administering the assessment remotely were aggregated and analyzed quantitatively. The relationship between BESS scores (overall and subscales) and ping, download and upload speeds were determined using Spearman correlation coefficients. To explore differences between operating systems on the remote BESS, the Mann–Whitney U test was used. Further, Spearman correlation coefficients were also used to examine overall technical error scores and these internet parameters. Statistical analyses were performed using IBM SPSS 27 statistical software.

## Results

A total of 26 undergraduate adults (mean age: 21.85 ± 2.95) were enrolled as part of the study. Our sample consisted of 54% female and 46% male and on average participants completed 14.50 (±0.86) years of education. Of the 26 participants, 13 received the BESS test remote first, and 13 received the BESS test in person first. On average each participant had 6.23 (±3.36) days between assessments. Number of days between assessments for those that took remote assessment first (mean: 5.31 ± 2.63) and those that took the in-person assessment first (mean: 7.15 ± 3.85) was not statistically significantly different (p = 0.17). 100% of participants indicated that their right leg was their dominant leg. Data on technical errors was recorded for 24 of the total participants. The most frequently used operating system was Windows (46.2%), and most participants took the assessment over Wi-Fi (92.3%). The metrics recorded for internet speeds showed no significant difference before and after the remote assessment (p > 0.05). The average ping of each participant was 15.59 ms (±7.56), download speed was 128.01 mbps (±157.4) and upload speed was 31.67 mbps (±67.72). There were no significant correlations found between technical errors and any of the specific internet speed parameters (all p > 0.05). Interrater reliability could only be calculated between raters 1 and 2, who completed most of the assessments together. Scores between raters had interclass correlation coefficients of 0.865 for the in-person assessment and 0.914 for the remote assessment, indicating a prominent level of agreement.

**Figure 1.**
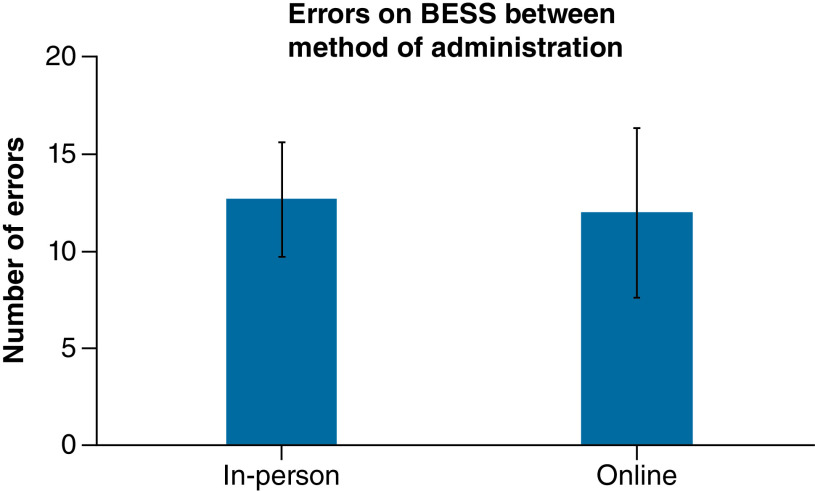
Bar graph of mean Balance Error Scoring System scores both online and in person. Error bars indicate standard deviation for each mean. Scores were similar with a p > 0.05. BESS: Balance Error Scoring System.

The scores for the in-person BESS assessment had a mean of 12.69 with a standard deviation of 2.96. The scores for the remote BESS assessment had a mean of 11.98 with a standard deviation of 4.38. There was no statistically significant (p = 0.312) difference between scores on the BESS test when administered remotely and in person (Figure 1). Additionally, overall scores between remote and in-person assessments were moderately correlated (rho = 0.563, p = 0.003)*.*

When comparing scores for each stance between modalities (remote vs in-person), they were comparable and demonstrated no significant difference (all p > 0.05) (Table 2).

**Table 2.** Mean scores between online and in person administrations of the Balance Error Scoring System.

Stance	Online	In-person	p-value
**Firm surface**			
Double leg stance	0.06 ± 0.22	0 ± 0	0.185
Single leg stance	2.63 ± 1.84	2.69 ± 1.66	0.876
Tandem leg stance	0.94 ± 1.11	1.19 ± 0.97	0.403
**Foam surface**			
Double leg stance	0.21 ± 0.43	0.15 ± 0.39	0.416
Single leg stance	4.87 ± 1.58	5.27 ± 1.14	0.198
Tandem leg stance	3.35 ± 1.41	3.38 ± 1.24	0.907
**Overall scores**	11.98 ± 4.38	12.69 ± 2.96	0.312

All p-values were nonsignificant (p > 0.05).

There was no correlation found between the number of technical errors and scores on the remote assessment (rho = 0.094, p = 0.663). Scores on the remote portion did not significantly correlate with download speed (rho = -0.073, p = 0.723), upload speed (rho = -0.303, p = 0.159) or ping (rho = -0.11,4 p = 0.580). There was no significant difference between remote BESS scores between Mac (n = 11) and Windows (n = 12) users (U = 35.000, p = 0.056).

## Discussion

This study investigated if scores on the BESS are altered when administered remotely. Scores were comparable between both modalities, suggesting that the reliability of BESS scoring remains consistent when extended to remote administration. Additionally, no significant differences were detected when comparing scores on individual stances.

There were certain minor difficulties observed with the remote assessment that are worthy of reporting, although they did not alter the results. Individuals being administered the BESS remotely are required to provide a spotter, which can complicate scheduling. When administering the in-person assessment, the rater is present to act as the spotter during the assessment, facilitating administration. If the remote assessment is extended to clinical use, test administrators may experience scheduling difficulties involving the presence of the spotter in the home setting. Other than the scheduling conflicts that emerged with the remote assessment, difficulties administering both modalities were minimal. Another difficulty was the poor camera quality of many laptop and desktop computers. While the poor quality did not affect the consistency of scores, it made certain scoring criteria difficult to assess. However, it is important to note that anecdotally, cell phone cameras had superior camera quality compared with laptops or desktops. Therefore, future studies may want to investigate the quality of mobile cameras compared with other cameras to determine whether any difference in camera quality improves telemedicine appointments.

There were several limitations with the study. The sample size and age of participants limits the generalizability of the findings. Future research investigating more diverse age groups may be warranted to expand generalizability. Additionally, our sample has extensive experience with remote services since many students were required to attend class over Zoom software when classes switched to remote during the pandemic. College-aged students may be more proficient with Zoom technology than other age groups. In future similar studies, it is suggested that researchers assess participants’ familiarity with Zoom or related video call technology. The present study also only investigated the consistency of scores for baseline use of the BESS test. For clinical applications, these results indicate that remote BESS administration can be used to obtain baseline scores. However, it may not be feasible to use remote administration for acute injury assessments because of the difficulty in providing the patient with the materials needed.

Overall, scores were comparable between modalities suggesting that the BESS can be administered remotely and still maintain its reliability. This finding yields several benefits. First, the remote version of the assessment may help improve access to individuals when performing the assessment in both clinical and research settings. Second, remote baseline assessments can generate a score that can be compared with an in-person postinjury assessment score. Based on these results, researchers and clinicians may benefit from remotely administering the BESS.

## Conclusion

This present study found that the BESS test maintains score consistency when administered remotely. Although technical issues did arise during administration, none greatly affected the ability of researchers to administer the test. These findings support the use of the BESS test as a remote tool, both clinically and for research purposes. This can increase access for both patients and research participants.

**Summary points**

- There is a lack of research into whether scores on the Balance Error Scoring System (BESS) are affected when administered remotely.
- This study had 26 undergraduate adults (mean age: 21.85 ± 2.95) taking the BESS remotely and in person.
- Half of the participants (n = 13) took the BESS remotely first and the remaining half (n = 13) took the BESS in person first.
- On an average, each participant had 6.23 (±3.36) days between assessments and the groups had no statistically significant difference in time between assessments (p > 0.05).
- The in-person BESS assessment scores had a mean of 12.69 with a standard deviation of 2.96 and the remote BESS assessment had a mean of 11.98 with a standard deviation of 4.38.
- There was no statistically significant difference between remote and in-person BESS scores (p > 0.05).
- This data indicates that reliability of the BESS test is not affected when administered remotely.
